# Nurses' Diet at Six Shillings a Head
*Last week an article appeared giving the Diet for a Week's Meals in Summer.


**Published:** 1912-08-31

**Authors:** 


### INSTITUTIONAL HOUSEKEEPING.

### Nurses' Diet at Six Shillings a Head.*

So much misapprehension exists even among experienced managers about the average cost per head of boarding women engaged in nursing that we think an alternative set of meals, arranged for winter, which can be provided at the above average cost will be welcomed by many matrons. It will strike everyone who examines these well thought-out menus that a good deal of variety is provided for the money. It must, of course, be well understood that the meals are subject to continual rearrangement, that winter is a wide term and admits many seasonal changes, and that they merely form a basis on which a clever housekeeper can build. They have the merit of being the result of actual practice and experiment instead of mere theory.

How then did the matron succeed in keeping down prices? This will be the first question of managers who would fain follow in her good track. To answer this question is to have a secure grasp on the household reins. But, while deprecating the notion that any complete answer can be given to it in the brief compass of one article, we would point out one or two ways in which economy was *not* arrived at. There was no skimping of either quality or quantity. No housekeeping can lay claim to efficiency or economy which cuts the workers short at every meal, or buys the coarsest variety of food obtainable, or keeps any end in view before that of satisfying natural appetites with wholesome victuals. Economy consists in the combination of innumerable pains and precautions converging to one end—the comfort of the household.

There is, however, one factor in economy which overpowers all the rest and may be said in great measure to account for the fine results achieved by good administrators in the well-being of their staff and the narrow figure at which this well-being works out in the accounts. Briefly, this consists in reserving all the expense for the food actually consumed, and spending nothing on food spoilt in the cooking, thrown away, left over on the plates, or wasted in other ways. The writer once had the privilege of visiting an enormous Poor-Law establishment at which the food for the staff worked out at an average of about 12s. a week per head, to the dissatisfaction of all the consumers. After inspecting the admirable kitchen premises, a request was made to see the larders, and the visitor was ushered into a little empty cupboard about 10 feet by 6, with two or three shelves. "And where do you put what is left over?" was the not unnatural remark. The reply was illuminating. "Nothing is ever left over." Now when a party of 200 or 300 persons sit down to a meal and the authorities are asked to believe and do believe that nothing is left over, it may be safely concluded that some one is doing pretty well out of the contracts for broken meats. It is not so very long since the bursar of one of the Cambridge colleges effected at one stroke an economy of £700 a year in the butcher's bill alone by the simple process of inquiring into the disposal of the joints left over from the high table.

We would ask our readers, then, to realise that the only possible method of feeding the nursing staff well and plentifully on six shillings a week is to take diligent heed that for every ten members of the household ostensibly boarded there are not enough good provisions wasted, thrown away or otherwise appropriated, to feed five more. It is not the food eaten and enjoyed which sends up the averages. The good housekeeper understands this and is never so happy as when appetites are sharp.

It is the countless, needless extravagances which take place in the store-cupboard and the kitchen. It is the excess paid for articles of common use by officials ignorant of market prices and placing undue confidence in contractors. It is the want of a woman thoroughly alert in the household, with time at her disposal to devote to economy.

* Last week an article appeared giving the Diet for a Week's Meals in Summer.

### A WEEK'S MEALS—WINTER.

| | BREAKFAST. | DINNER. | SUPPER. |
|---|---|---|---|
| Sunday: | Sausages. Marmalade. | Cold Roast Pork. Potatoes. Beetroot. Date Pudding, Sweet Sauce. Oranges. | Cold Beef. Baked Potatoes. Cheese. Biscuits. Butter. Coffee. |
| Monday: | Fried Bacon. Watercress. | Steak Pudding. Stewed Figs. Custard. | Fried Fish and Potatoes. Macaroni. Cheese. Coffee. |
| Tuesday: | Tongue. Golden Syrup. | Roast Mutton. Two Vegetables. Apple Tart. | Shepherd's Pie. Tapioca Pudding. Cheese. Coffee. |
| Wednesday: | Porridge. Kedgeree. | Irish Stew. Haricot Beans. Potatoes. Currant Pudding. Apples. | Cold Meat. Pickles. Potatoes. Gingerbread. Cheese. Coffee. |
| Thursday: | Ham or Potted Meat. | Roast Beef. Two Vegetables. Treacle Tart. | Soup. Stewed Fruit. Custard. Cheese. Coffee. |
| Friday: | Scrambled Eggs. Marmalade. | Fish. Potatoes. Jam Pudding. | Hot-Pot. Blancmange. Coffee. |
| Saturday: | Dried Fish. Porridge. | Boiled Pork. Pease Pudding. Potatoes. Macaroni Pudding. | Fish Pie. Jam Tart. Coffee. Cheese. |
| | Tea and Coffee. Toast. Butter. Brown and White Bread. | If preferred: Cold Meat. Milk Pudding. | Cold Meat, if preferred. Milk or Cocoa, if preferred. |
| | TEA. Cake. Jam. Fruit on alternate days. | Milk. Lemonade. Hot Water. Ale, if liked. | |

